# The Effect of Testing Turnaround Time on COVID-19 Outbreak Severity Within U.S. Nursing Homes

**DOI:** 10.1093/geroni/igab046.2705

**Published:** 2021-12-17

**Authors:** Annie Rhodes, Leland Waters, Faika Zanjani, Tracey Gendron, Rick Moore

**Affiliations:** Virginia Commonwealth University, Richmond, Virginia, United States

## Abstract

COVID-19 has brought renewed attention to infectious diseases in U.S. nursing homes (NHs). The Testing turnaround time (TAT) of SARS-CoV-2 is vital information, supporting staff ability to make decisions regarding resource allocation.

**Methods:**

Using data obtained from the National Healthcare Safety Network’s COVID-19 nursing home data set, we analyzed the TAT of laboratory polymerase chain reaction (PCR) testing on outbreak severity (number of people infected) for residents and staff. A MANOVA was performed on NHs submitting data over 26 weeks (May-November 2020). The independent variable was the average TAT for the two weeks prior (<24 hours, 1-2 days, 3-7 days, or 7+ days).

**Results:**

N = 15,363 NHs. The TAT for the combined dependent variables of staff and resident COVID-19 cases. F(10,781,354) = 3161.265, Pillai’s trace = .078, p<.0005, partial η2=.4. The average outbreak severity for staff was 13.93 cases when TAT was < 24 hours, compared to 15.29 cases at 1-2 days. For residents, the difference was less pronounced but still significant. The average outbreak severity for residents was 17.07 cases when TAT was<24 hours, compared to 18.61 cases when the TAT was 1-2 days. Tukey post-hoc tests found significance for all levels of testing for residents and staff at p<.0005.

**Discussion:**

Time differences to receive PCR test results from a laboratory are significant in outbreak severity for staff and residents. The most meaningful result positively impacting the ultimate spread and severity of gross cases is when the TAT for PCR results is < 1 day.

